# Recruitment of racial and ethnic minorities to clinical trials conducted within specialty clinics: an intervention mapping approach

**DOI:** 10.1186/s13063-018-2507-9

**Published:** 2018-02-17

**Authors:** Rossybelle P. Amorrortu, Mariana Arevalo, Sally W. Vernon, Arch G. Mainous, Vanessa Diaz, M. Diane McKee, Marvella E. Ford, Barbara C. Tilley

**Affiliations:** 1 Department of Biostatistics & Data Sciences, The University of Texas Health Science Center at Houston (UT Health) School of Public Health, 1200 Hermann Pressler, Houston, TX 77030 USA; 2 Department of Health Promotion and Behavioral Sciences, The University of Texas Health Science Center at Houston (UT Health) School of Public Health, 1200 Hermann Pressler, Houston, TX 77030 USA; 3 Department of Health Services Research Management and Policy, Health Science Center, University of Florida College of Public Health and Health Professions, PO Box 100195, Gainesville, FL 32610 USA; 4 Department of Family Medicine, Medical University of South Carolina, 5 Charleston Center Dr., Suite 263, Charleston, SC 29425 USA; 5 Department of Family and Social Medicine, Albert Einstein College of Medicine, 1300 Morris Park Avenue Block, Room 417, Bronx, NY 1046 USA; 6 Department of Public Health Sciences and Hollings Cancer Center, Medical University of South Carolina, 68 President Street, Suite BE103, Charleston, SC 29425 USA

**Keywords:** Program development, Trust, Relationship building, Social cognitive theory, Specialist, Training

## Abstract

**Background:**

Despite efforts to increase diversity in clinical trials, racial/ethnic minority groups generally remain underrepresented, limiting researchers’ ability to test the efficacy and safety of new interventions across diverse populations. We describe the use of a systematic framework, intervention mapping (IM), to develop an intervention to modify recruitment behaviors of coordinators and specialist investigators with the goal of increasing diversity in trials conducted within specialty clinics. To our knowledge IM has not been used in this setting.

**Methods:**

The IM framework was used to ensure that the intervention components were guided by health behavior theories and the evidence. The IM steps consisted of (1) conducting a needs assessment, (2) identification of determinants and objectives, (3) selection of theory-informed methods and practical applications, (4) development and creation of program components, (5) development of an adoption and implementation plan, and (6) creation of an evaluation plan.

**Results:**

The intervention included five educational modules, one in-person and four web-based, plus technical assistance calls to coordinators. Modules addressed the intervention rationale, development of clinic-specific plans to obtain minority-serving physician referrals, physician-centered and patient-centered communication, and patient navigation. The evaluation, a randomized trial, was recently completed in 50 specialty clinics and is under analysis.

**Conclusions:**

Using IM we developed a recruitment intervention that focused on building relationships with minority-serving physicians to encourage minority patient referrals. IM enhanced our understanding of factors that may influence minority recruitment and helped us integrate strategies from multiple disciplines that were relevant for our audience.

## Background

Racial and ethnic minority groups (American Indians and Alaska Natives, Asians, Hawaiian or Pacific Islanders, African Americans or Blacks, and Hispanics) in the USA are disproportionately affected by a variety of health conditions such as type II diabetes mellitus, cardiovascular disease, stroke, HIV/AIDS, and some types of cancer [1, 2]. Yet recent studies in various diseases (*e.g.*, cancer, neurologic diseases, and cardiovascular disease) indicate that study populations underrepresent the race and ethnicity of those affected by these diseases [3, 4]. The limited inclusion of diverse racial and ethnic groups in clinical trials hinders the ability to test efficacy and safety of new treatments and limits the generalizability of findings to a broader population [5, 6]. This circumstance is a major limitation as the minority population in the USA increases and health disparities in treatment advances may widen for minority populations [7].

Previous research identified minority patient barriers to participation in clinical trials such as mistrust in medical researchers, fear of randomization/experimentation, and other logistical issues (language, transportation and financial barriers) [8–12]. Researchers have also reported facilitators to participation such as altruism, perceived personal benefit, good patient-provider relationship and increased awareness about available trials [9]. However, less is known about the efficacy of recruitment strategies. A systematic review examined the literature on minority recruitment interventions from 1966 to 2005 and concluded that community outreach as a single recruitment strategy may be insufficient [13]. The authors emphasized the need for researchers to identify evidenced-based strategies to increase enrollment of minorities in trials. A recent review of minority recruitment interventions addressing multiple levels of recruitment barriers found that strategies such as opening trials within community sites to overcome transportation problems, and the use of nurse navigators and research staff to overcome specific patient barriers helped increase minority recruitment [14]. The authors also identified the importance of having investigators develop trusting relationships with community clinicians in clinical trial recruitment efforts. In further review of the literature we found that trust plays a critical role in recruitment efforts. Trust between patients, clinicians, and investigators can influence patient referrals, willingness, decisions to participate, and adherence to treatment protocols [15, 16], but there is a paucity of interventions focusing on building trust to enhance trial recruitment. While promising strategies have been identified, there has been little rigorous testing in randomized trials. The development of novel interventions that address trust as a critical component are required to determine the best approaches to increase racial and ethnic minority recruitment.

A previous, unsuccessful study [17] employed a rigorous randomized trial designed to evaluate a minority recruitment intervention aimed at building trusting relationships with minority-serving physicians; however, the investigators did not use a systematic framework such as intervention mapping (IM) [18] to develop the intervention. To address the need for more rigorous and evidenced-based minority recruitment interventions, we used IM to develop the intervention to be tested in the Randomized Recruitment Intervention Trial (RECRUIT). The intervention was designed to modify recruitment behaviors of coordinators and specialist investigators, with emphasis on building trusting relationships with referring physicians, with the goal of increasing racial and ethnic diversity in multi-site clinical trials conducted within specialty clinics. This paper describes the six steps of the IM framework used to develop the intervention. To our knowledge, this is the first IM-guided intervention program designed to increase minority recruitment into trials that has targeted specialist investigators and coordinators.

## Methods

IM is a systematic framework that uses theory and evidence to develop behavior change interventions. IM is an iterative process that includes six steps [18]. The first step is to conduct a needs assessment and a review of the literature to identify the scope of the problem. The second step consists of selecting behavioral determinants and specifying performance objectives. The third step consists of selecting theory-informed methods and practical applications. The fourth step consists of brainstorming, outlining, producing, and pre-testing program components and materials. The fifth step consists of ensuring adoption and implementation of the intervention program. The sixth and final step consists of creating an evaluation plan to assess the expected program outcomes.

## Results

### Step 1: needs assessment

In September 2011, we received funding for RECRUIT and formed a planning group consisting of 14 stakeholders including specialist investigators and family practice physicians from diverse racial and ethnic backgrounds, and an interdisciplinary team including a psychologist, epidemiologist, biostatistician, and behavioral scientists. We used the prior experience of our planning group to guide the development of the intervention. As a first step we conducted a needs assessment, drawing information from (a) a literature review, (b) two advisory groups, and (c) findings from two previous projects:

#### ***Literature review (a)***

Our review of recruitment strategies found that successful community-based approaches that included strategies such as mass mailings and community outreach, have been developed for recruiting minority populations for common diseases such as diabetes mellitus and hypertension [19, 20]; however, few have been rigorously tested using comparative research designs. More important, we found few studies of minority recruitment to trials of less common diseases, particularly those requiring physician referrals to specialty care [21]. Such trials are often conducted at specialty clinics where minority patients often have limited access to services [22–24]. These findings suggested the need for recruitment strategies that facilitate the process of referrals from physicians outside of the specialty clinics who may see a higher proportion of minority patients.

Next, we examined the literature on barriers and facilitators to physician referrals of patients to specialty clinics [25, 26] and of coordinators because they also play a pivotal role in enrolling patients into the trials [14]. This review identified specialist investigator and coordinator-level determinants that could influence their abilities to enroll diverse participants into clinical trials, such as communication barriers with minority patients, limited skills to encourage external referrals of minority patients, beliefs about patient participation, attitudes about minority participation in trials, and time constraints [24, 27–29]. We found that barriers to referrals from the referring physicians’ perspective included lack of awareness about open trials, mistrust in the medical institutions and investigators conducting trials, and concerns of losing patients to the specialist investigators [30–32]. From literature on patients’ perspectives, trust emerged as a barrier to enrollment in trials across diverse racial and ethnic groups [11]. Additionally, the literature suggests that patients are more likely to enroll in a trial if asked by their personal physician [9, 33]. Together, these findings identified the primary recruitment strategy to be encouraged at the specialty clinics requiring referrals where our study was conducted, *i.e.*, relationship-building to generate referrals from minority-serving physicians.

#### ***Advisory groups (b)***

Meetings were conducted with existing community advisory groups from the Medical University of South Carolina Hollings Cancer Center and the Centers for Disease Control and Prevention-funded REACH 2010 diabetes project to further explore barriers and facilitators to minority recruitment. The advisory groups suggested that specialist investigators should build trusting personal relationships with local minority-serving physicians. Specifically, the advisory groups stated that specialist investigators should be encouraged to personally meet with minority-serving physicians to discuss the trial and develop a mutually beneficial relationship. They suggested further training for specialist investigators and coordinators, stressing the importance of respectful listening in physician-patient interactions, and recommended use of the term “medical research” because the term “clinical trial” is generally not well-understood and may carry a negative connotation among community members. We used these findings to guide the intervention training content.

#### ***Findings from two previous projects (c)***

In a study funded by the National Institute of Neurologic Disorders and Stroke, to increase participant diversity in a randomized controlled trial of Parkinson’s disease, the investigators conducted in-depth post-trial interviews with specialist investigators (*n* = 24) and coordinators (*n* = 24) to explore enrollment barriers [17]. Challenges to recruitment included lack of flexibility in recruitment methodology, insufficient understanding of the definition of ethnic and racial groups, the use of inappropriate outreach strategies (*e*.*g*., recruiting through support groups that had low minority membership), and lack of recruitment staff and bilingual resources within the specialty clinics [17]. Lessons learned from this study included the need to allow each clinic to customize their recruitment approach based on their needs, and to provide each clinic with clear definitions of minority groups being targeted for recruitment. In a second qualitative study, six focus groups were conducted to elicit potential solutions to commonly reported barriers to clinical trial participation from African American (*n* = 32) and Hispanic (*n* = 25) participants [34]. Findings from this study suggested that increasing physician-patient trust by training physicians on how to communicate about clinical trials to diverse audiences, providing participant incentives, prioritizing participant convenience, and utilizing patient navigation interventions could improve minority participation in trials. Findings from these two formative studies guided the planning group’s decisions for the development of the intervention.

Throughout our needs assessment, trust continually emerged as an important component in developing relationships between specialty investigators and minority-serving physicians and between minority patients and their providers. The planning group conceptualized these findings as a trust triangle dynamic whereby enrollment would be facilitated by increasing trust between patients, referring physicians, and specialist investigators [35]. The planning group used information gathered from the literature and prior research to develop a logic model that described the problem of low enrollment of minorities in trials [36]. The model outlined predisposing, enabling, and reinforcing determinants of the target behavior, *i.e.,* recruitment of minority participants, which is published elsewhere [35].

### Step 2: selecting behavioral determinants and performance objectives

The planning group used findings from step 1 to select determinants that could influence specialty investigators and coordinators’ behavior to increase the number of minorities enrolled in clinical trials. They selected determinants from social cognitive theory (SCT) [37], which posits that human behavior is influenced by personal, cognitive and environmental factors. According to SCT, individuals are more likely to adopt a new behavior if they have a sense of self-efficacy, gain knowledge and skills to overcome obstacles, and have positive expectations about adopting a new behavior [37]. Thus, the planning group selected the following determinants for specialist investigators and coordinators: knowledge, skills, outcome expectations, and self-efficacy for enrolling minority participants into trials.

After the planning group identified the determinants, they specified the performance objectives for the behavioral outcomes, *i.e.*, those behaviors that specialty investigators and coordinators would have to perform in order to make the desired behavioral changes. For example, in order for specialty investigators to work effectively with minority-serving physicians and their staff, the specialty investigators would have to:

- Make a plan to approach potential minority-serving physicians in their community
- Develop strategies for establishing trusting relationships with minority-serving physicians
- Develop an ongoing system of communication with minority-serving physicians regarding their patients and
- Discuss potential roles for referring minority-serving physicians regarding their referred patients (*e.g*., whether the referring-minority-serving physician wanted to continue to manage their patient’s health care)

### Step 3: selecting theory-informed intervention methods and practical applications

After the planning group identified the SCT behavioral determinants in step 2, they selected theory-informed intervention methods and practical applications to influence the determinants. Theory-informed intervention methods are techniques derived from behavioral theory to influence changes in the SCT determinants. Based on the literature, the planning group chose methods from the following theories: theory of information processing [38], social cognitive theory [37], goal setting theory [39], and the trans-theoretical model [40]. Table 1 provides examples of the theoretical methods that were chosen from these theories.

**Table 1:** Intervention social cognitive theory (SCT) determinants, methods and practical applications

SCT determinants	Theoretical methods	Practical applications
Knowledge	• Discussion^a^	• Discussion with specialist investigators and coordinators at in person meeting and subsequent webinars
	• Elaboration^a^	• Specialist investigators prompted to discuss clinical experiences relating to differences in outcomes by race and ethnicity
Skills and self-efficacy	• Modeling^b^	• Videos
	• Skills training^b^	• In person meeting • Continuous quality improvement (CQI) training • Group work: mapping recruitment process and completion of fishbone diagram • Web-based educational modules (communication, navigation, CQI)
	• Feedback^c^	• Reports (on screening, recruitment and patient satisfaction) • Individual follow-up calls • Group calls with other sites
	• Goal setting^c^	• Minority recruitment plan
	• Verbal persuasion^b^	• Videos • Individual follow-up calls
	• Active learning^b^	• Individual follow-up calls • Videos • Web-based educational modules
	• Persuasive communication^b^	• Presentations at in-person meeting • Web-based educational modules
	• Individualization^d^	• Individual follow-up calls
Outcome expectations	• Modeling^b^	• Videos • Group calls with other sites
	• Active learning^b^	• In-person meeting • Web-based educational modules

^a^Theory of information processing

^b^Social cognitive theory

^c^Goal setting theory

^d^Trans-theoretical model

Next, they identified practical applications, or ways that theoretical methods were applied in an intervention in a manner suitable for its population and context [18]. The planning group made decisions about practical applications to implement these methods. For example, we provided specialist investigators and coordinators with technical assistance via conference calls. We also adapted approaches from continuous quality improvement (CQI) and patient navigation (PN). CQI is a familiar approach for people who work in healthcare settings, and has been shown to be effective in improving healthcare processes [41, 42]. CQI methodology can be used to flowchart current practices and identify ways to improve processes to achieve the desired outcome, an increase in minority-serving physician referrals [43]. PN programs are oriented toward problem solving to help patients address and overcome barriers to care rather than delivering a predefined set of services. PN has been used by investigators and coordinators to increase accrual of minority patients into clinical trials [44], but this strategy has not been tested as a part of a randomized trial. Table 1 provides examples of practical applications for each behavioral determinant.

### Step 4: producing program components and materials and pre-testing

During this step, the planning group developed and refined a draft of the intervention scope, sequence, and structure. The planning group decided to deliver the intervention in a flexible and accessible manner because our target audience (*i.e.,* specialist investigators and coordinators) were part of multi-site trials that had locations across the USA. Thus, they selected a web-based/teleconference delivery method combined with one initial in-person meeting. Next, the planning group outlined the content of educational modules and other program materials using findings from step 1 to select the key topics to be addressed in the modules. For example, issues addressed included the importance of diversity in clinical trials, development of a minority recruitment plan, and strategies to overcome recruitment barriers. The intervention was designed to be applicable to trials conducted in specialty clinics by promoting the use of physician referrals as a primary recruitment strategy regardless of the disease or condition under study. The planning group worked in small teams to develop educational modules in their area of expertise.

The planning group then pre-tested the final intervention components during an in-person meeting paying particular attention to feedback from specialist investigators and family practice physicians, to help ensure that the intervention was relevant to the target audience. For example, the planning group incorporated educational modules on physician-to-physician communication and patient communication. The behavioral scientists and psychologist on the planning group suggested that these educational modules include training in the skill of active listening. The clinicians suggested use of physicians and coordinators rather than actors in the videos demonstrating communication strategies.

### Description of the intervention components and delivery method

The final intervention included five educational modules, individual follow-up calls with coordinators, and group calls, as needed (Table 2). The first module was an in-person initiation meeting (“kick off”). One or more specialist investigators and coordinators from each specialty clinic were invited to attend. During this meeting, participants learned about the importance of minority inclusion in trials and building trusting relationships for the purpose of recruitment. They also learned to use CQI strategies to tailor minority recruitment plans specific to their clinic needs. As seen in Table 2, some of the module elements could be customized to the disease conditions under study at their clinics.

**Table 2:** Intervention scope and sequence

Module	Module 1	Module 2	Module 3	Module 4	Module 5
Month	1	2	3	4	5
Delivery method	In person	Webinar	Webinar	Webinar	Webinar
Title	*Kick-off* *meeting*	*Working with referring Physicians*	*CQI: developing a* *minority recruitment plan*	*Communicating with* *participants*	*Participant navigation* *(coordinators only)*
Content	*Presentation:* a brief overview of the RECRUIT intervention^a^ ᅟ *Discussion:* disparities in morbidity/mortality of disease^a^ ᅟ *Presentation:* importance of minority recruitment: the NIH and FDA perspective ᅟ *Presentation:* importance of minority participation: biologic aspects^a^ ᅟ *Discussion:* differences in treatment outcomes for minorities ᅟ *Presentation:* what we know and do not know about minority recruitment ᅟ *Presentation:* importance of trust and relationship building in recruiting minority patients ᅟ *Presentation:* introduction to continuous quality improvement (CQI) for minority recruitment ᅟ *Activity:* flowchart of current recruitment process ᅟ *Activity:* fishbone diagram of low referrals from minority serving physicians	*Activity:* CQI updates and potential physician referral sources by site representative (SI or coordinator) ᅟ *Presentation:* barriers to referrals and physician-centered communication ᅟ *Video and discussion:* Physician-to-physician communication video	*Activity:* site representative (SI or coordinator) present their fishbone diagram ᅟ *Activity:* site representative (SI or coordinator) share their site’s CQI minority recruitment plan	*Activity:* site representative (SI or coordinator) discusses CQI update ᅟ *Presentation:* recap on communicating with minority-serving physicians ᅟ *Presentation:* patient-centered communication ᅟ *Video and discussion:* patient communication ᅟ *Video and discussion:* Participant’s fear of experimentation (“guinea pig”)	*Activity:* Coordinators discuss CQI updates ᅟ *Discussion:* take-home activity on communication styles ᅟ *Presentation:* how can coordinators become recruitment navigators? ᅟ *Video and discussion:* participant navigation ᅟ *Discussion:* what is currently done at your site and what could be added to help patients overcome structural barriers to recruitment
Take home activity	Schedule a meeting with the rest of the trial team and review material from the meeting Revise fishbone if needed ᅟ Finding your patients worksheet ᅟ CQI minority recruitment plan worksheet ᅟ Review map of minority-dense areas within their communities to identify minority-serving physicians	Revise minority recruitment plan ᅟ SIs to make personal contact with at least two potential referring minority-serving physicians	Revise and implement your minority recruitment plan	Develop Q&A sheet for potential participants about enrolling in the trial ᅟ Continue to implement and revise your minority recruitment plan ᅟ Coordinators: communications style assessment (optional)	Navigation resources worksheet

*NIH National Institutes of Health, FDA Food and Drug Agency, SI* specialist investigator, *Q&A* question and answer

^a^Content can be customized to the condition under study by the clinical trial

Modules 2–5 were delivered through web conferencing software. These 1-h web-based modules addressed topics related to barriers to obtaining minority-serving physician referrals, approaches to physician-centered communication, refinement of their clinic-specific plans to improve minority recruitment, patient-centered communication, and patient navigation. Specialist investigators and coordinators were invited to attend modules 2–4; attendance by specialist investigators at module 5 was optional. In module 5, coordinators learned to apply patient navigation strategies to participant recruitment. This module focused on teaching coordinators to provide emotional, informational, and instrumental support to facilitate participant enrollment into trials.

After each module, a 30-min CQI follow-up call was held with each specialty clinic coordinator and a CQI coach, who could provide encouragement, technical assistance and problem-solving on any continuing clinic-specific barriers to minority recruitment. The first CQI call occurred 2 weeks after module 1 and included the clinic’s specialist investigator. The specialist investigator was encouraged to attend the first call to ensure that the clinic was working to develop and implement a minority recruitment plan tailored to their clinic needs. Subsequent individual CQI calls were scheduled monthly with coordinators until the end of the trial recruitment period or for 2 years, whichever occurred first. Two months after clinics completed module 5, a group CQI call was scheduled where specialist investigators and coordinators shared successful strategies and lessons learned from their recruitment experience. Additional group calls were scheduled as needed.

### Step 5: developing an adoption and implementation plan

The next step was to identify adopters and implementers of the intervention. Adopters are individuals or organizations that can decide to adopt an intervention and implementers are individuals who are responsible for delivering the intervention. The planning group designed the intervention with the idea that the adoption would occur in a two-step process. First, the leadership at a multi-site clinical trial coordinating center would decide to adopt the intervention then the site investigators would agree to adopt it. The implementation would also occur in a 2-step process. First, staff at the multi-site coordinating center would be the implementers in charge of delivering training for the intervention program. Next, specialty investigators and coordinators at each site who participate in the intervention program would subsequently tailor the acquired information and recruitment strategies to their site needs before implementing them. Because the intervention was being developed and tested, members of the planning group acted as the step-1 intervention implementers. The multi-site coordinating center only had to give permission for the planning team to contact the sites. The planning group developed an implementation plan outlining the tasks associated with delivery of the intervention components (*e.g*., modules on communication, CQI calls, kick-off meeting materials), and met regularly to revise ways to deliver the intervention. To ensure the implementation plan was ready and covered all aspects of intervention delivery, the planning group conducted several intervention practice sessions and refined the content of the modules, the activities, and the delivery sequence.

### Step 6: creating an evaluation plan

The final task was to develop an evaluation plan including the study design, sample size, data sources, data collection procedures, and a data analysis plan. Members of the planning group designed a cluster randomized trial to assess the intervention outcomes within 60 specialty clinics. Specialty clinics were recruited from multi-site clinical trials studying a variety of conditions and interventions. Those clinics accepting participation in the RECRUIT trial were randomized to the intervention or control group. A detailed description of the trial design is provided elsewhere [35]. The trial primary outcome was the proportion of minorities enrolled in the intervention and control clinics, and was assessed from minority recruitment data gathered from the participating clinical trial coordinating centers. We also evaluated specialist investigators and coordinators’ self-efficacy and outcome expectations, and screening and recruitment activity logs to record the number of screened patients, reasons for patient refusal, and number of interactions between specialist investigators and minority-serving physicians. For process evaluation we monitored program attendance rates and the quality of intervention delivery. We collected survey data about patient satisfaction with their experience at study visits and their interactions with the specialist investigators and coordinators. In addition, qualitative in-depth interviews were conducted with specialist investigators and coordinators to understand more about the strengths and limitations of the intervention and what could be improved. The trial evaluating the intervention was conducted from 2013 to 2017 with 50 specialty clinics from four national clinical trials. Process and outcome evaluation data are not yet available, but final outcomes will be reported in a subsequent manuscript.

## Discussion

To our knowledge, this is one of the first theory-informed minority recruitment interventions to be rigorously tested within multi-site clinical trials in various disease areas. Several recruitment interventions that target trial investigators and research staff conducting clinical trials are reported in the literature; however, we only identified two randomized trials [17, 21] and a single study that used IM to incorporate theoretical components in their intervention [45], unlike ours, which uniquely incorporates both.

A major component of our intervention was an in-person kick-off meeting with specialist investigators and coordinators. Although other researchers have used similar training sessions for physician investigators, trial managers, and trial coordinators, their intervention “away days,” which provided additional training in study procedures and encouraged discussions about recruitment strategies across sites did not increase the number of patients screened, consented, or randomized into an orthopedic surgical trial [46]. In addition, their intervention was informally assessed, and the authors acknowledged the need to formally evaluate their intervention in a randomized trial. As a way to possibly strengthen their intervention, the authors discussed the inclusion of stakeholders from the target population as members of their planning committee. In contrast, our intervention was guided by a planning group that included specialist investigators to ensure the appropriateness and relevance of the intervention methods to specialist investigators and coordinators conducting trials within specialty clinics.

There is a published educational intervention to increase accrual of older persons to a cooperative group of cancer trials that targeted physicians and research team members and was rigorously evaluated using a randomized design [21]. However, findings suggest that the intervention did not have an impact on participant accruals into trials within the study time period. The authors concluded that changing physician behavior was a difficult task, and without addressing reinforcing and enabling factors it was harder to impact behavior. In our experience, IM helped us identify a theoretical framework and theory-informed methods that could influence behavior change. We anticipate that this will positively impact our intervention outcome. Additionally, the intervention by Kimmick and colleagues [21] targeted one trial investigator and one other research team member from each institution in the cooperative group, with the idea that these two would share the training information with the other cooperative group researchers conducting other studies at their institution. The authors mentioned that limiting the intervention audience may have hampered the dissemination of their intervention at the institution. On the other hand, our intervention was designed to target trial-specific specialist investigators and coordinators from each specialty clinic within a single multi-site clinical trial. We expected that by intervening at this individual trial level our intervention could be more specifically tailored to the trial and reach our target population of specialist investigators and coordinators.

In 2012, a group of researchers conducted a randomized study to evaluate whether building trusting relationships with minority-serving community physicians increased referrals of minority participants to a Parkinson’s disease trial [17]. The primary intervention was to host continuing medical education events for local minority-serving physicians with presentations by experts who were to be role models for the minority clinicians. Their intervention also included one didactic training session for physician investigators and follow-up group calls with the coordinators. Their intervention was not effective, and the investigators concluded that it was difficult to build trusting relationships with referring physicians through a single event and that there was little engagement from investigators to foster these relationships. Building on the experience from this trial, our planning group developed our intervention to provide ongoing training on CQI methods for specialist investigators and coordinators to develop and implement a site-specific minority recruitment plan. We provided more encouragement for specialist investigators to take an active role in relationship building with minority-serving physicians by training specialist investigators to reach out in person to local physicians. We also included coordinators in our intervention and provided coordinator-focused participant navigation training to overcome potential barriers faced by minority participants.

Recently, researchers used IM to develop an intervention to increase participation in HIV clinical trials as part of a community-based research study, called Project Education and Access to Services and Testing (EAST) [45]. The Project EAST intervention helped community physicians locate potential clinical trials and refer their patients to them. The authors’ experience using IM was similar to ours in that they found that it was beneficial to use health behavior theories, formative research and stakeholders’ perspectives to develop their intervention. However, unlike the rigorous evaluation design of RECRUIT, we could not find a published description of their planned evaluation.

Based on our experience developing the intervention, we found IM to be a time-consuming and resource-intensive process. We estimate that the planning group, consisting of a multidisciplinary investigative team, spent 1 year carrying out IM steps 1–3, and developing plans for program development and evaluation. After funding, the team spent an additional 1.5 years of work developing the intervention components and pilot testing, excluding the time it took them to carry out the evaluation. This time limitation has been encountered by others [45, 47]. We further estimate that implementation of our program by a multi-site coordinating center would require some effort from a coordinating center investigator and at least one recruitment coordinator who would deliver the training of our program to the specialist investigators and coordinators at the participating specialty clinics. Also of note, the total number of recruitment coordinators needed is dependent upon the number of sites participating in the trial. Additionally, the specialist investigators and coordinators working at the participating specialty clinics would need to devote some effort to implement the intervention strategies, such as making contacts with minority-serving physicians.

## Conclusions

Regardless of the RECRUIT trial results, using this comprehensive and systematic approach we developed a recruitment intervention that focused on building relationships with minority-serving physicians to encourage minority patient referrals. IM helped us to enhance our understanding of the factors that influence minority recruitment, select appropriate theory-informed intervention methods to address these factors, and integrate strategies from multiple disciplines that were relevant to our target audience. From a practical standpoint, not many program planners, practitioners, or trialists are familiar with the IM process, which could help develop or adapt existing interventions. This description of our approach will familiarize other investigators with the process and evidence-based strategies we used in developing our trust-based minority recruitment program. We hope that our experience will encourage others to use IM to adapt our minority recruitment intervention to their needs, or develop their own intervention program.

## Abbreviations

CQI: Continuous quality improvement
EAST: Education and Access to Services and Testing
IM: Intervention mapping
PN: Patient navigation
RECRUIT: Randomized Recruitment Intervention Trial
SCT: Social cognitive theory
